# FBXO10 prevents chronic unpredictable stress‐induced behavioral despair and cognitive impairment through promoting RAGE degradation

**DOI:** 10.1111/cns.13727

**Published:** 2021-09-07

**Authors:** Jiacen Li, Qingcui Zeng, Wenjie Su, Menglong Song, Min Xie, Lei Mao

**Affiliations:** ^1^ Department of Anesthesiology Sichuan Provincial People’s Hospital University of Electronic Science and Technology of China Chengdu China; ^2^ Chinese Academy of Sciences Sichuan Translational Medicine Research Hospital Chengdu China; ^3^ Geriatric Intensive Care Unit Sichuan Provincial People’s Hospital University of Electronic Science and Technology of China Chengdu China; ^4^ Emergency Intensive Care Unit Sichuan Provincial People’s Hospital University of Electronic Science and Technology of China Chengdu China

**Keywords:** chronic unpredictable stress, depression, FBXO10, microglia, RAGE

## Abstract

**Aims:**

Depression is one of the leading causes of disability worldwide. The receptor for advanced glycosylation end products (RAGE) is closely related to chronic stress and is a target of F‐box protein O10 (FBXO10) which promotes the degradation of RAGE by ubiquitination. Here, we explored the role of FBXO10 and RAGE in chronic unpredictable stress (CUS)‐induced behavioral despair, cognitive impairment, neuroinflammation, and the polarization microglia.

**Methods:**

Male C57BL/6 mice with or without infusion of viral in the medial prefrontal cortex (PFC) were subjected to CUS. Then the mice were exposed to forced swim test, sucrose consumption test, novelty‐suppressed feeding test, and temporal object recognition task to assess the behavioral despair and cognitive impairment. Inflammatory cytokines and the neurotrophic factor brain‐derived neurotrophic factor (BDNF) levels in PFC were assessed by enzyme‐linked immunosorbent assay. Immunofluorescence and immunohistochemistry staining were performed to observe the activation and phenotypic transformation of microglia in PFC. LPS‐induced cell model was constructed to explore the effect of FBXO10/RAGE axis in the polarization of microglia *in vitro*.

**Results:**

FBXO10 promoted RAGE degradation by ubiquitination in BV2 cells. FBXO10 protein levels were reduced whereas RAGE protein levels were enhanced in CUS mice. FBXO10 overexpression or RAGE knockdown inhibited proinflammatory cytokine release, promoted BDNF expression, mitigated the depressive‐like and cognitive impairment behaviors, and affected the polarization of microglia induced by CUS exposure. FBXO10/RAGE axis promoted the polarization of microglia from the M1 to the M2 phenotype *in vitro*. Moreover, p38 MAPK and NF‐κΒ were identified to be the downstream effect factors for FBXO10/RAGE axis.

**Conclusions:**

FBXO10 administration prevents CUS‐induced behavioral despair, cognitive impairment, neuroinflammation, and the polarization of microglia through decreasing the accumulation of RAGE, p38 MAPK, and NF‐κΒ, suggesting potential therapeutic strategies for the prevention and treatment of depression.

## Graphical abstract

FBXO10 promoted RAGE degradation by ubiquitination. FBXO10/RAGE axis is involved in chronic unpredictable stress‐induced behavioral despair, cognitive impairment, and neuroinflammation and regulates the polarization of microglia in vitro and in vivo

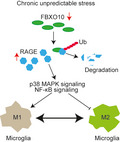

## 1 INTRODUCTION

Depression is a major source of disability that causes great social and economic burdens^1^, ^2^ and it is urgent for understanding molecular and cellular mechanisms that drive the pathophysiology of depression. Exposure to chronic stress is an important risk factor for depression, which could decrease dendrite branching and spine density.^3^, ^4^ The chronic unpredictable stress (CUS) model provides the possibility to explore neurobiological regulation mechanisms, which could promote microglia‐mediated neuronal remodeling and synapse loss in the medial prefrontal cortex (PFC) and associated behavioral consequences.^2^, ^5^ These results suggest that neuron‐microglia interactions contribute to PFC dysfunction. The potential mechanisms of functional changes in microglia induced by chronic stress remain to be further explored nevertheless.

The receptor for advanced glycosylation end products (RAGE) is a membrane‐bound receptor and expressed by neurons, glia, and endothelial cells.^6^ RAGE affects neuronal differentiation, cell survival, neurite outgrowth, and neuroinflammation by binding to different ligands.^7^ RAGE signaling pathway was also reported strongly associated with depression. The stimulation of RAGE in Aβ‐enriched conditions activates JNK and p38MAPK which further promotes IL‐1β release and affects synaptic depression.^8^ Exposure to stress activates RAGE signaling and downstream NF‐κΒ signaling to increase transcription of the proinflammatory cytokine, which further promotes depressive behaviors.^9^ The interaction between advanced glycation end products (AGEs) and RAGE induces upregulation of mitogen‐activated protein kinases (MAPKs) and NF‐κB, leading to endothelial dysfunction.^10^However, the role of RAGE in depressive still needs more exploration.

F‐box protein O10 (FBXO10) is one of F‐box components possessing ubiquitin E3 ligase activity.^11^, ^12^ In human lymphoma, FBXO10 binds antiapoptotic protein BCL‐2 and accelerates its degradation.^11^ FBXO10 functions as a potential tumor suppressor in human lymphoma cells.^13^ FBXO10 binds to human germinal center‐associated lymphoma (HGAL), leading to the ubiquitylation and degradation of HGAL, which is involved in lymphomagenesis and other immune processes.^14^ However, the effect of FBXO10 on depression is rarely reported. Importantly, FBXO10 also is found to interact with RAGE and accelerates its degradation.^12^ It may be inferred that FBXO10 may be involved in depression, given the close association between RAGE and depression.

Thus, we investigate the effects of FBXO10/RAGE axis in regulating microglial polarization and its role in CUS. Our results revealed that M1 phenotype microglial was increased in the medial PFC after CUS exposure and FBXO10/RAGE axis could promote microglial polarization from the M1 phenotype to the M2 phenotype *in vitro* and *in vivo*, which may provide potent therapeutic strategies for depression.

## 2 MATERIALS AND METHODS

### 2.1 Animals

Male C57BL/6 mice (8–12 weeks old) were purchased from Charles River (Beijing, China) and group‐housed (4/cage) in 11.5” ×7.5” ×6” polypropylene cages under a 12 h light–dark cycle with ad libitum access to water and rodent chow. For all studies, mice were randomly assigned to experimental groups. All animal protocols followed the guidelines established by the National Institutes of Health and were approved by the Animal Care and Use Committee of the Sichuan Provincial People's Hospital.

### 2.2 Chronic unpredictable stress (CUS)

CUS was performed as previously described.^2^ In brief, mice were exposed to random stressors daily for 14 days. Stressors included cage rotation, isolation, physical restraint, radio noise, food or water deprivation, rat odor, light on overnight, light off during day, stroboscope overnight, crowding, wet bedding, no bedding, or tilted cage. The detailed procedure about the CUS was shown in Table S1.

### 2.3 Surgery and cortical infusion

Mice were anesthetized with ketamine/xylazine (100/10 mg/kg). Bilateral viral infusions in the medial PFC (1 μL; 0.1 μL/minute) were performed with coordinates (from bregma), +2.0 mm anterior‐posterior, ±0.2 mm medial‐lateral, and −2.8 mm dorsal‐ventral.^2^ Incisions were closed with sutures and mice received intraperitoneal injection of carprofen (5 mg/kg) immediately after surgery and daily for the next two days.

### 2.4 Behavioral testing

Forced swim test (FST), sucrose consumption test (SCT), novelty‐suppressed feeding test (NSF), and temporal object recognition task (TOR) were conducted as previously described.^2^ Behavioral testing was performed in lieu of stressor on day 12 (FST), day 13 (SCT), day 14 (NSF), and day 15 (TOR) of the experimental paradigm. All behavioral tests were carried out at 08:00–11:00 in a normally lit room (white light). Mice were exposed to all behavior/cognitive tests prior to sacrifice for brain region dissection. Immunofluorescence and immunohistochemistry staining were performed on paraffin brain sections as previously described.^15^ Data were analyzed with ImageJ (NIH Image, Bethesda, MD, USA) to calculate the fluorescence intensity or counting number of recognized cells per field. The details are provided in Supplemental materials.

### 2.5 Cell culture

BV2 cell line were obtained from Procell (Wuhan, China) and maintained in Roswell Park Memorial Institute (RPMI) medium 1640 supplemented with 10% fetal bovine serum and antibiotics (penicillin 100 U/mL, streptomycin 100 μg/mL). Infected cells were exposed to lipopolysaccharide (LPS, 100 ng/mL) for 16 h to induce the polarization of microglia.

### 2.6 Flow cytometry

To determine the cell quantity of different phenotypes after indicated treatment, harvested cell suspensions were incubated Alexa Fluor^®^ 488 mouse monoclonal antibody to CD86 (Abcam, ab256270) and Alexa Fluor^®^ 488 rabbit monoclonal antibody to CD206 (Abcam, ab195191). Cells were washed, re‐suspended in FACS buffer, and detected by flow cytometer (CytoFLEX S, Beckman Coulter, USA). Non‐specific binding was assessed using isotype‐matched antibodies.

### 2.7 Co‐immunoprecipitation

Total protein (500 μg) from cell lysates was precleared with 40 μL protein A/G beads for 30 min. Then, primary antibody was added for incubation at 4℃ overnight. Beads were slowly centrifuged, washed, and heated at 100℃ for 5 min with 80 μL of protein sample buffer before SDS‐PAGE and immunoblotting.

### 2.8 Statistical analysis

Results are expressed as the means ± SEM. The normality of the data distribution was analyzed by the Shapiro‐Wilk test. To compare differences between two groups, normally distributed continuous variables were compared by Student's t‐test, while non‐normally distributed variables were compared by the Mann‐Whitney test. For multiple comparisons among three groups or more groups, data were analyzed using one‐way analysis of variance (ANOVA) followed by Bonferroni's post hoc test if the data were normally distributed or by the Kruskal‐Wallis test if the data were non‐normally distributed. A *P* value of <0.05 was considered to indicate a significant difference. All analyses were performed by GraphPad Prism 8 software (GraphPad Software, La Jolla, CA, USA).

The detailed information for viral preparation, Western blot, RNA isolation and real‐time PCR, ELISA, Protein Half‐Life Detection, and *in vitro* ubiquitination assays are provided in Supplemental materials.

## 3 RESULTS

### 3.1 FBXO10 targets RAGE for ubiquitination and degradation in BV2 cells

**FIGURE 1:**
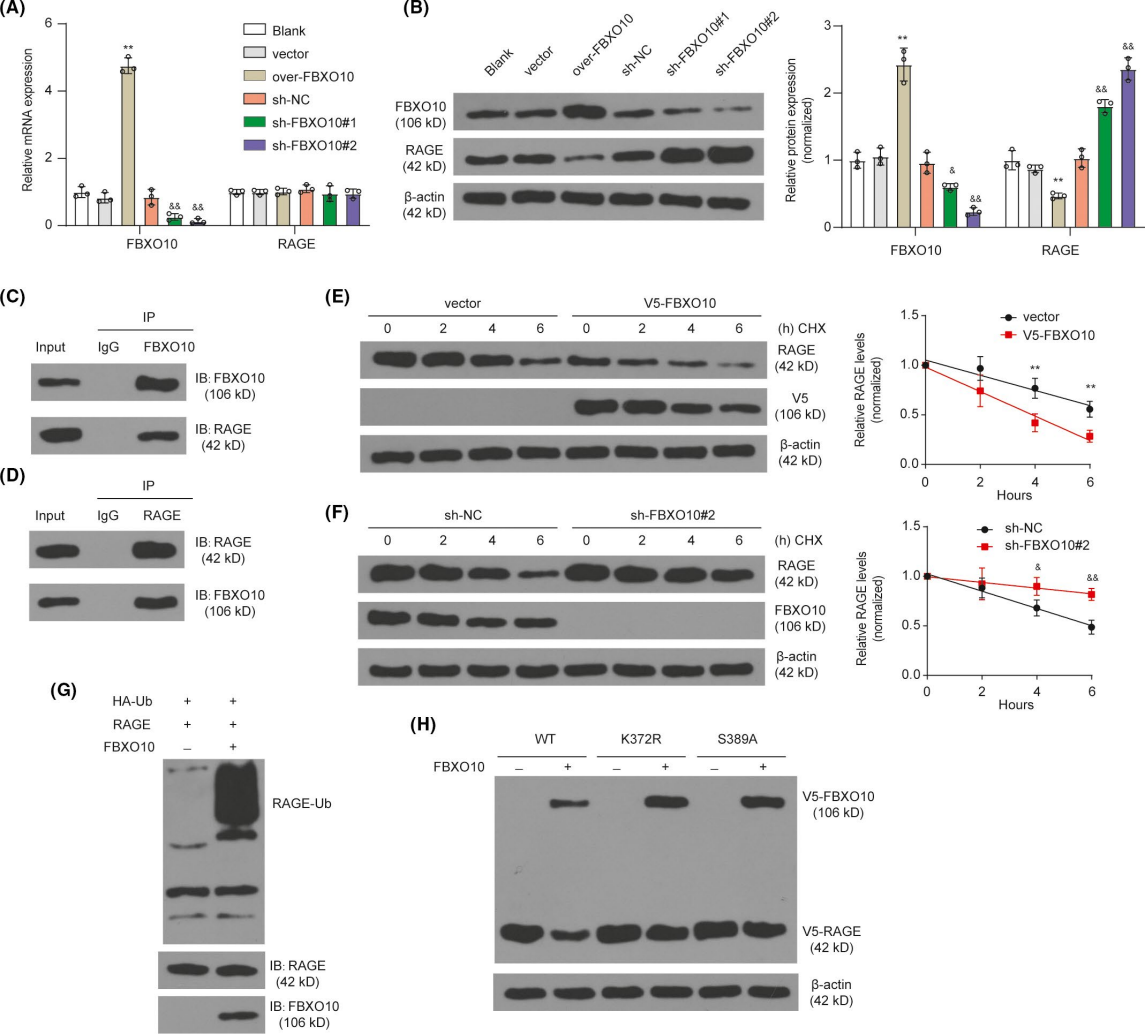
FBXO10 regulates RAGE degradation in BV2 cells. (A) The mRNA levels of FBXO10 and RAGE in BV2 cells after FBXO10 overexpression or knockdown were detected by qRT‐PCR. **P < 0.01 vs. vector; &&*P *< 0.01 vs. sh‐NC; n = 3 per group. (B) The protein levels of FBXO10 and RAGE in BV2 cells after FBXO10 overexpression or knockdown were detected by Western blotting. **P < 0.01 vs. vector; &&*P *< 0.01 vs. sh‐NC; n = 3 per group. (C) The interaction between RAGE and FBXO10. IP for FBXO10 and immunoblotting for FBXO10 or RAGE. (D) IP for RAGE and immunoblotting for FBXO10 or RAGE. (E) BV2 cells were transfected with 1 µg empty vector or 1 µg V5‐FBXO10 for 72 h and then treated with CHX (100 µg/ml) for 2, 4, or 6 h. **P < 0.01 vs. vector; n = 3 per group. (F) BV2 cells were transfected with scrambled shRNA or FBXO10 shRNA for 72 h and then treated with CHX (100 µg/ml) for 2, 4, or 6 h. &*P *< 0.05, &&*P *< 0.01 vs. sh‐NC; n = 3 per group. (G) RAGE ubiquitination assays using BV2 cells transfected with the indicated expression vectors. (H) FBXO10 co‐overexpression reduces RAGE WT but not RAGE K372R and RAGE S389A levels. Cells were transfected with RAGE WT, RAGE K372R, or RAGE S389A, with or without FBXO10 plasmid, and immunoblotted for V5

Considering that RAGE was highly conserved in human and mouse and the interaction between FBXO10 and RAGE in Beas‐2B cells, we wondered whether FBXO10 regulates RAGE level in BV2 cells. We firstly altered FBXO10 expression by a recombinant FBXO10 overexpression vector or two shRNA for FBXO10. As shown in Figure 1A and B, the mRNA and protein level of FBXO10 was upregulated after the transfection of FBXO10 overexpression vector. The mRNA and protein level of FBXO10 was severally inhibited by the both two shRNA for FBXO10. Notably, RAGE protein level was reduced after FBXO10 overexpression and enhanced after FBXO10 knockdown (Figure 1B). Exogenous changes in FBXO10 expression had no effect on RAGE mRNA levels. Furthermore, co‐immunoprecipitation experiments indicated a strong interaction between RAGE and FBXO10 protein (Figure 1C and D). FBXO10 overexpression increased RAGE degradation in CHX chase experiments (Figure 1E). FBXO10 knockdown inhibited the degradation of RAGE induced by CHX stimulation (Figure 1F). Considering the direct interaction between FBXO10 and RAGE and FBXO10 is an authentic E3 ligase subunit, we next examined whether FBXO10 directly regulates the ubiquitination of RAGE. FBXO10 overexpression enhanced the levels of ubiquitinated RAGE (Figure 1G). K374 and S391 are reported to be the key molecular sites for RAGE stability,^12^ which are conserved in human and mouse (K372 and S389). We next co‐overexpressed FBXO10 in cells with RAGE constructs (WT, K372R, S389A). FBXO10 overexpression decreased V5‐RAGE levels when co‐transfected with RAGE WT plasmid, but not with the K372R and S389A plasmid (Figure 1H). In short, these results indicated that FBXO10 promoted RAGE degradation by ubiquitination partly dependent on K372 and S389 residues of RAGE.

### 3.2 FBXO10/RAGE axis participates in CUS

**FIGURE 2:**
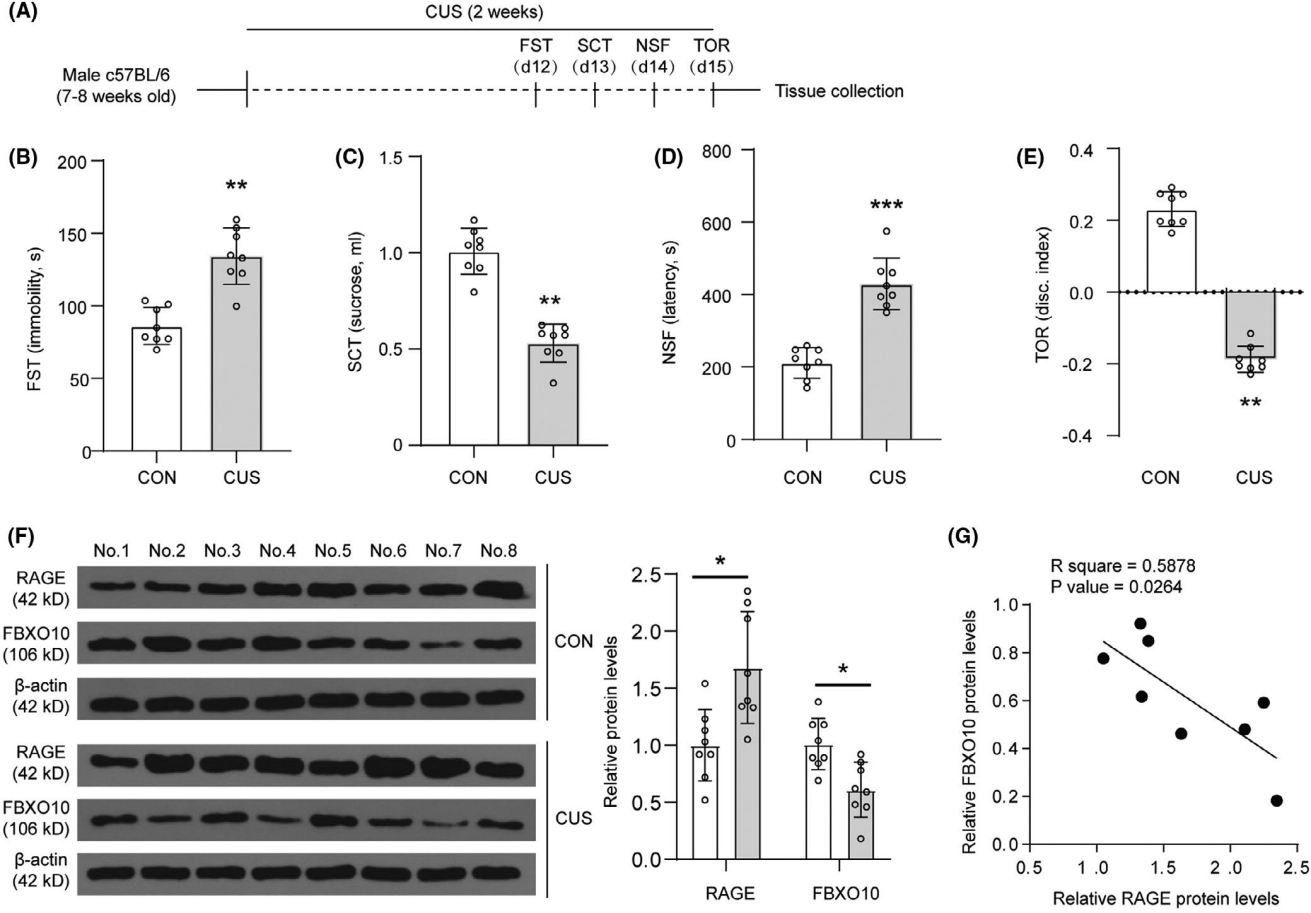
FBXO10/RAGE axis is involved in CUS. (A) Schematic depicting experimental approach and timeline. (B) Immobility in the forced swim test (FST) was shown (n = 8 per group). (C) Total sucrose consumer was shown (n = 8 per group). (D) Latency to feed in the novelty‐suppressed feeding test (NSF) was shown (n = 8 per group). (E) Discrimination index in the temporal object recognition (TOR) task was shown (n = 8 per group). (F) The protein levels of RAGE and FBXO10 in PFC were detected by Western blotting (n = 3 per group). (G) Correlation analysis for RAGE and FBXO10 protein levels in PFC. Bars represent the mean ±SEM. *P < 0.05, **P < 0.01, ***P < 0.001 vs. control mice (CON)

Considering the interaction between FBXO10 and RAGE in BV2 cells and the strong association between RAGE and depression, we speculated that FBXO10/RAGE axis may play a role in depression. To confirm this conjecture, we then detected the expression of FBXO10 and RAGE in CUS mice. Male mice were exposed to 2 weeks of CUS and then assessed in the FST, SCT, NSF, and TOR on subsequent days (Figure 2A). In the FST, mice exposed to CUS had increased immobility (Figure 2B). The sucrose consumption was decreased in CUS compared to controls mice (Figure 2C). Similarly, there was a general increase in latency to feed in the NSF following CUS (Figure 2D). Furthermore, CUS caused significant cognitive impairment with reduced discrimination index in the TOR (Figure 2E). Thus, chronic stress‐induced behavioral and cognitive consequences were observed after CUS. In addition, we found that FBXO10 protein levels were reduced whereas RAGE protein levels were enhanced in the medial PFC of CUS mice (Figure 2F). It is worth noting that FBXO10 protein levels were negatively correlated with RAGE protein levels in CUS mice (Figure 2G). Taken together, these studies showed that FBXO10/RAGE axis may participate in CUS.

### 3.3 CUS‐induced behavioral despair, cognitive impairment, and neuroinflammation are prevented by FBXO10 overexpression

**FIGURE 3:**
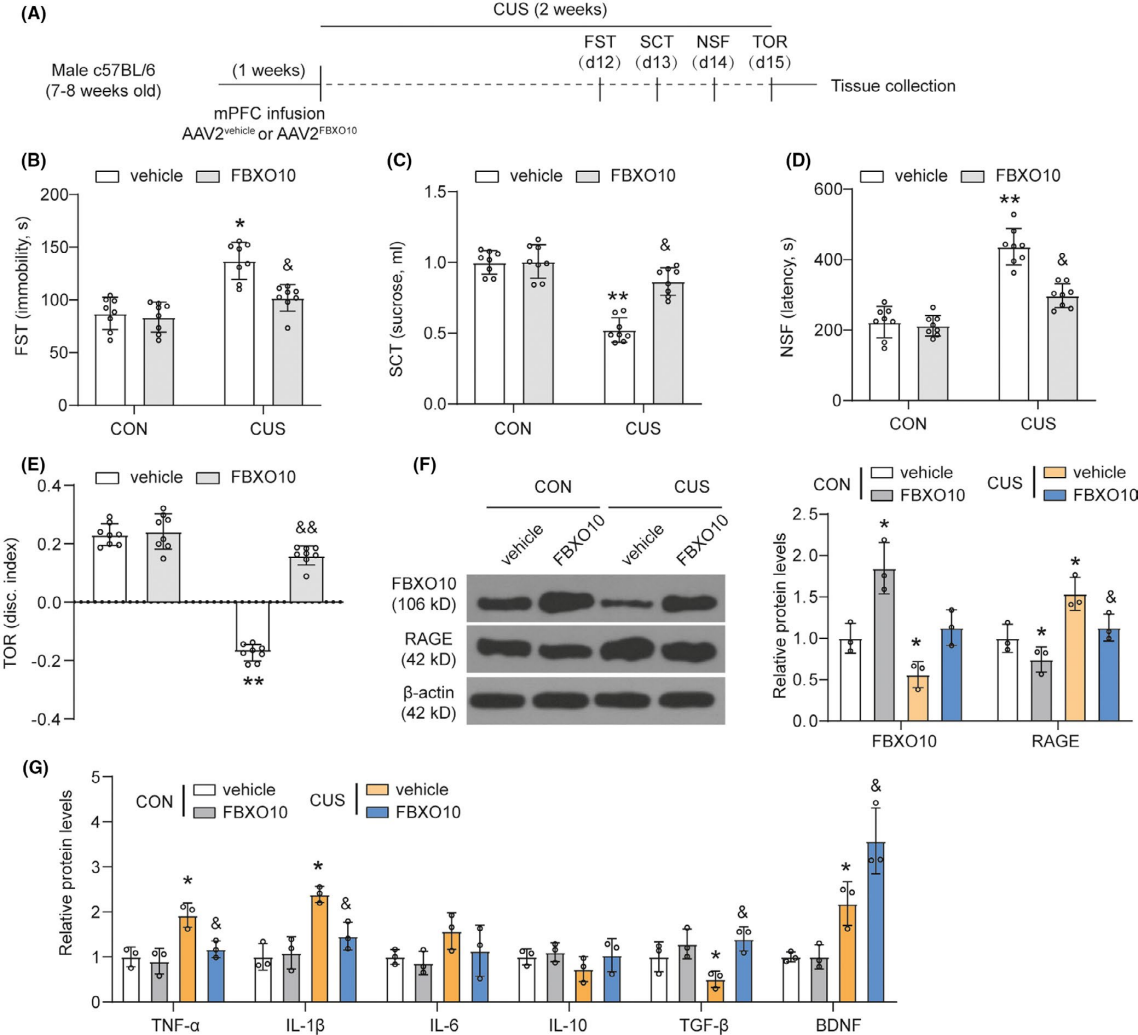
CUS‐induced behavioral despair, cognitive impairment, and neuroinflammation are prevented by FBXO10 overexpression. (A) Schematic depicting experimental approach and timeline. Male wild‐type mice received bilateral infusion of AAV2^vehicle^ or AAV2^FBXO10^ and were subjected to 14 days of CUS after recovery. (B‐E) Immobility in the FST, total sucrose consumer, latency to feed in the NSF, and discrimination index in the TOR were shown (n = 8 per group). (F) The protein levels of RAGE and FBXO10 in PFC were detected by Western blotting (n = 3 per group). (G) The levels of inflammatory cytokines TNF‐α, IL‐1β, IL‐6, IL‐10, TGF‐β, and the neurotrophic factor brain‐derived neurotrophic factor brain‐derived neurotrophic factor (BDNF) in tissue homogenate of medial PFC were measured using ELISA (n = 3 per group). Bars represent the mean ±SEM. *P < 0.05, **P < 0.01 vs. AAV2^vehicle^ control mice; &*P *< 0.05, &&*P *< 0.01 vs. AAV2^vehicle^ CUS mice

After viral packaging, C57BL/6 male mice received bilateral infusion of AAV2^vehicle^ or AAV2^FBXO10^ in the medial PFC. Mice were then subjected to CUS after recovery (Figure 3A). FBXO10 overexpression prevented the CUS‐induced increase in immobility, decrease in sucrose consumption, increase in latency to feed in the NSF, and decrease in discrimination index in the TOR (Figure 3B‐E). AAV2^FBXO10^ infusion enhanced FBXO10 protein levels in controls and CUS mice (Figure 3F). Reduced RAGE protein levels were found in the medial PFC of AAV2^FBXO10^ CUS mice. These data showed that FBXO10 overexpression prevented CUS‐induced behavioral despair and cognitive consequences.

Neuroinflammation plays a vital role in the development of depressive behavior,^16^ we next detected the release of proinflammatory cytokine. The results indicated that CUS mice had a higher level of TNF‐α and IL‐1β compared to the controls mice, and FBXO10 overexpression inhibited TNF‐α and IL‐1β expression induced by CUS (Figure 3G). CUS mice had a lower level of TGF‐β. FBXO10 overexpression enhanced TGF‐β level in CUS mice. However, CUS did not significantly affect the expression of IL‐6 and IL‐10 in the medial PFC. Besides, the neurotrophic factor brain‐derived neurotrophic factor (BDNF), which is beneficial for neuronal survival and brain repair, was also found to be upregulated in CUS mice and FBXO10 overexpression further enhanced BDNF levels in the medial PFC of CUS. These findings indicate that CUS exposure significantly altered the expression of neuroinflammation, and FBXO10 overexpression could partially reverse these changes.

### 3.4 CUS‐induced behavioral despair, cognitive impairment and neuroinflammation are prevented by RAGE knockdown

**FIGURE 4:**
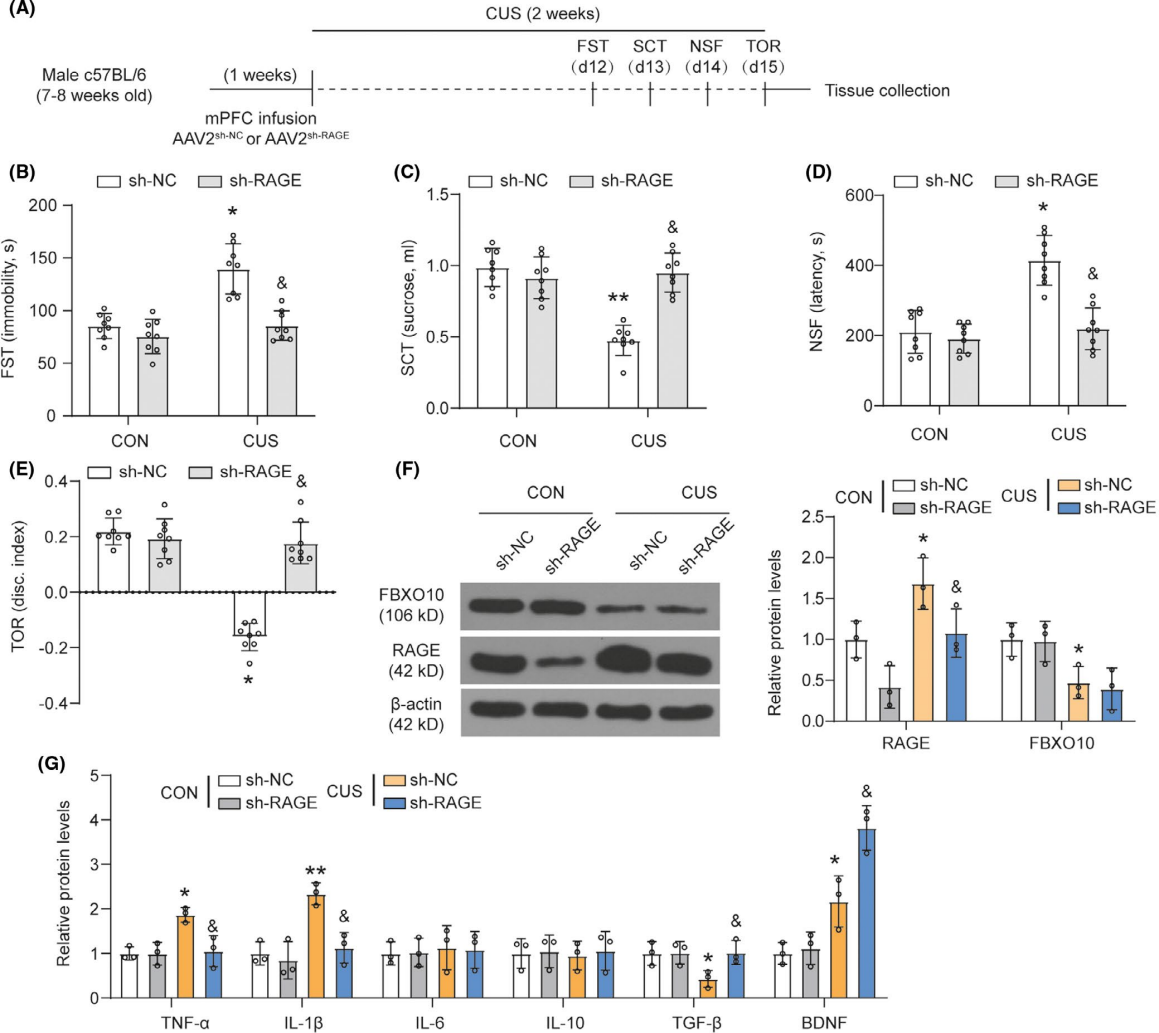
CUS‐induced behavioral despair, cognitive impairment, and neuroinflammation are prevented by RAGE knockdown. (A) Schematic depicting experimental approach and timeline. Male wild‐type mice received bilateral infusion of AAV2^sh−NC^ or AAV2^sh−RAGE^ and were subjected to 14 days of CUS. (B‐E) Immobility in the FST, total sucrose consumer, latency to feed in the NSF, and discrimination index in the TOR were shown (n = 8 per group). (F) The protein levels of RAGE and FBXO10 in PFC were detected by Western blotting (n = 3 per group). (G) The levels of TNF‐α, IL‐1β, IL‐6, IL‐10, TGF‐β, and BDNF in tissue homogenate of medial PFC were measured using ELISA (n = 3 per group). Bars represent the mean ±SEM. *P < 0.05, *P < 0.01 vs. AAV2^sh−NC^ control mice; &*P *< 0.05 vs. AAV2^sh−NC^ CUS mice

C57BL/6 male mice received bilateral infusion of AAV2^sh−NC^ or AAV2^sh−RAGE^ in the medial PFC (Figure 4A). Similar to FBXO10 overexpression, RAGE knockdown prevented the CUS‐induced increase in immobility, decrease in sucrose consumption, increase in latency to feed in the NSF, and decrease in discrimination index in the TOR (Figure 4B‐E). RAGE knockdown reduced RAGE protein levels in the medial PFC in both controls and CUS groups and there was no significant change in the expression of FBXO10 after RAGE knockdown (Figure 4F). These findings indicated that RAGE knockdown prevented CUS‐induced behavioral despair and cognitive consequences. We also found that RAGE knockdown inhibited TNF‐α and IL‐1β expression induced by CUS and promoted TGF‐β and BDNF expression in CUS groups (Figure 4G). However, RAGE knockdown did not significantly affect the expression of IL‐6 and IL‐10 in the medial PFC. These findings suggest that RAGE knockdown could partially reverse neuroinflammation induced by CUS.

### 3.5 FBXO10 prevented CUS‐induced behavioral despair, cognitive impairment, and neuroinflammation by promoting RAGE degradation

**FIGURE 5:**
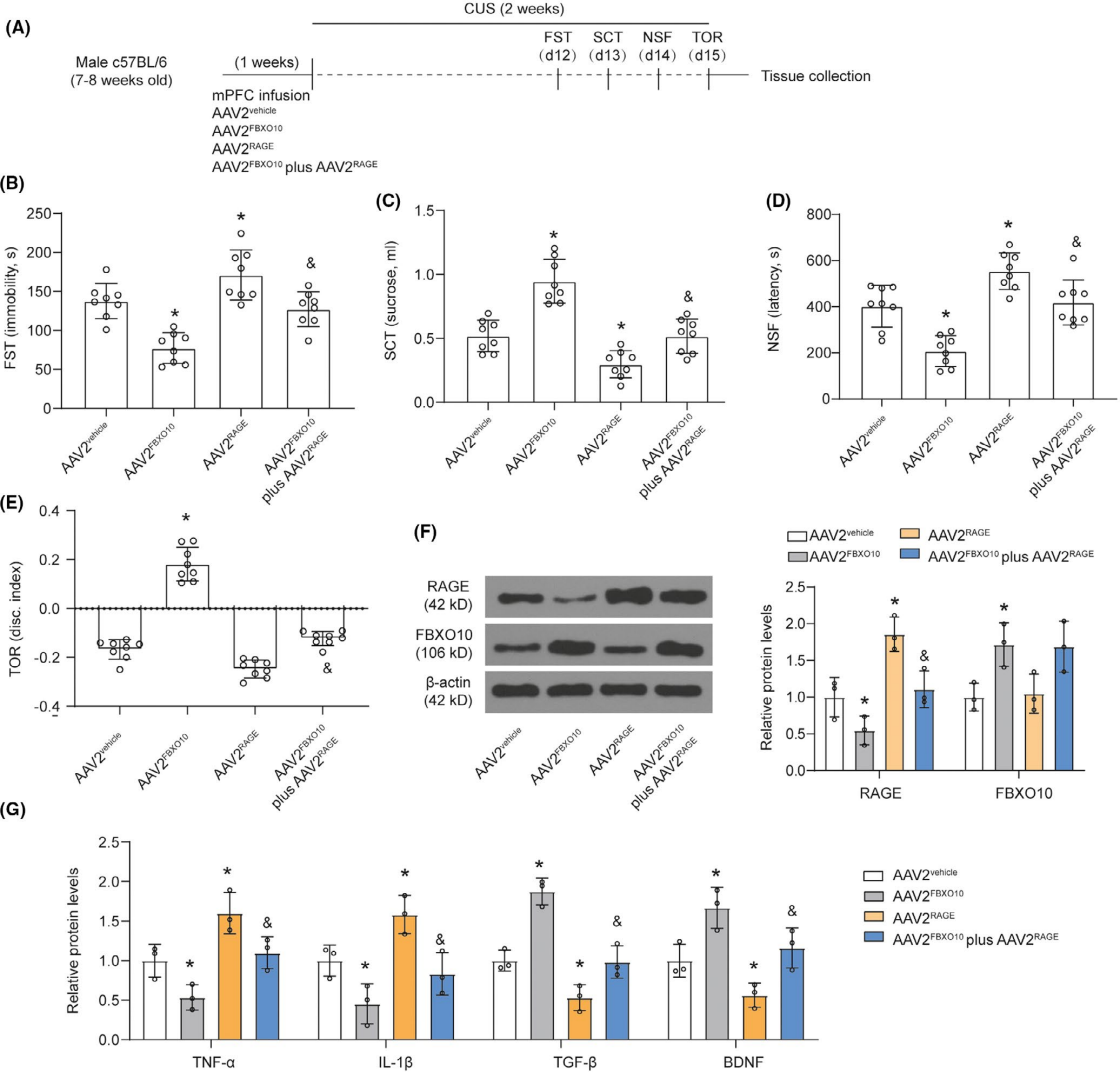
FBXO10 prevented CUS‐induced behavioral despair, cognitive impairment, and neuroinflammation by promoting RAGE degradation. (A) Schematic depicting experimental approach and timeline. Male wild‐type mice received bilateral infusion of AAV2^vehicle^, AAV2^FBXO10^, AAV2^RAGE^, or AAV2^FBXO10^ plus AAV2^RAGE^ and were subjected to 14 days of CUS. (B‐E) Immobility in the FST, total sucrose consumer, latency to feed in the NSF, and discrimination index in the TOR were shown (n = 8 per group). (F) The protein levels of RAGE and FBXO10 in PFC were detected by Western blotting (n = 3 per group). (G) The levels of TNF‐α, IL‐1β, TGF‐β, and BDNF in tissue homogenate of medial PFC were measured using ELISA (n = 3 per group). Bars represent the mean ±SEM. *P < 0.05 vs. AAV2^vehicle^; &*P *< 0.05 vs. AAV2^FBXO10^

C57BL/6 male mice received bilateral infusion of AAV2^vehicle^, AAV2^FBXO10^, AAV2^RAGE^, or AAV2^FBXO10^ plus AAV2^RAGE^ in the medial PFC (Figure 5A). RAGE overexpression reversed the increase in immobility, decrease in sucrose consumption, increase in latency to feed in the NSF, and decrease in discrimination index in the TOR induced by FBXO10 overexpression in CUS mice (Figure 5B‐E). Consistent with the previous experimental results, FBXO10 overexpression decreased RAGE levels, and RAGE overexpression did not affect FBXO10 levels in the medial PFC of CUS mice (Figure 5F). However, RAGE overexpression rescued RAGE levels reduced by FBXO10 overexpression. We also found that RAGE overexpression rescued TNF‐α, IL‐1β, TGF‐β, and BDNF expression induced by FBXO10 overexpression (Figure 5G). These results suggest that FBXO10 prevents CUS‐induced behavioral despair, cognitive impairment, and neuroinflammation by promoting RAGE degradation.

### 3.6 FBXO10/RAGE axis regulates morphological alterations of microglia in the medial PFC after CUS exposure

**FIGURE 6:**
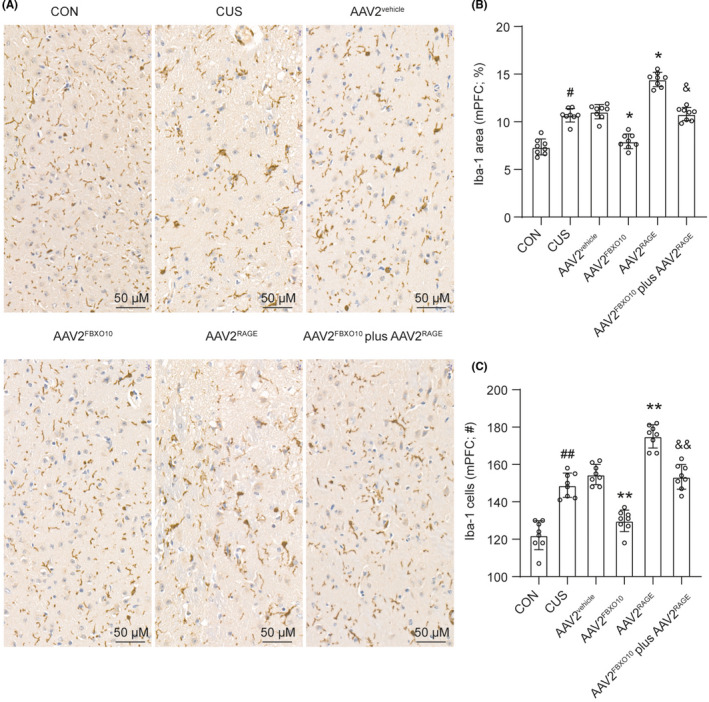
FBXO10/RAGE axis regulates morphological alterations in microglia in the medial PFC after CUS exposure. Male wild‐type mice received bilateral infusion of AAV2^vehicle^, AAV2^FBXO10^, AAV2^RAGE^, or AAV2^FBXO10^ plus AAV2^RAGE^ and were subjected to 14 days of CUS. Three hours after the final stressor, mice were perfused and brains collected for immunohistology. (A) Representative images of Iba‐1 immunohistochemistry in the medial PFC were shown. Scale bar represents 20 μm. (B) Quantification of Iba‐1 proportional (%) area in the medial PFC (n = 8 per group). (C) Number of Iba‐1 microglia per field in the medial PFC (n = 3 per group). Bars represent the mean ±SEM. #*P *< 0.05, ##*P *< 0.01 vs. control mice; *P < 0.05, **P < 0.01 vs. AAV2^vehicle^; &*P *< 0.05, &&*P *< 0.01 vs. AAV2^FBXO10^

We next explored the morphological alterations in microglia and Iba‐1 was selected as a microglia marker. Representative images of Iba‐1 immunohistochemistry in control or CUS mice with indicated treatment were shown in Figure 6A. CUS exposure significantly increased Iba‐1 positive microglia in the medial PFC (Figure 6B). FBXO10 overexpression reduced the density of Iba‐1 positive microglia and RAGE overexpression enhanced the density of Iba‐1 positive microglia in the medial PFC. Besides, RAGE overexpression rescued the density of Iba‐1 positive microglia in the medial PFC induced by FBXO10 overexpression. Proportional area analyses of Iba‐1 showed that CUS significantly increased Iba‐1 immunolabeling in the medial PFC (Figure 6C), indicating morphological changes in microglia. These results suggest that CUS exposure promotes microglia activation, and FBXO10/RAGE axis was involved in CUS‐induced microglia activation.

### 3.7 FBXO10/RAGE axis regulates the polarization of microglia in the medial PFC after CUS exposure

**FIGURE 7:**
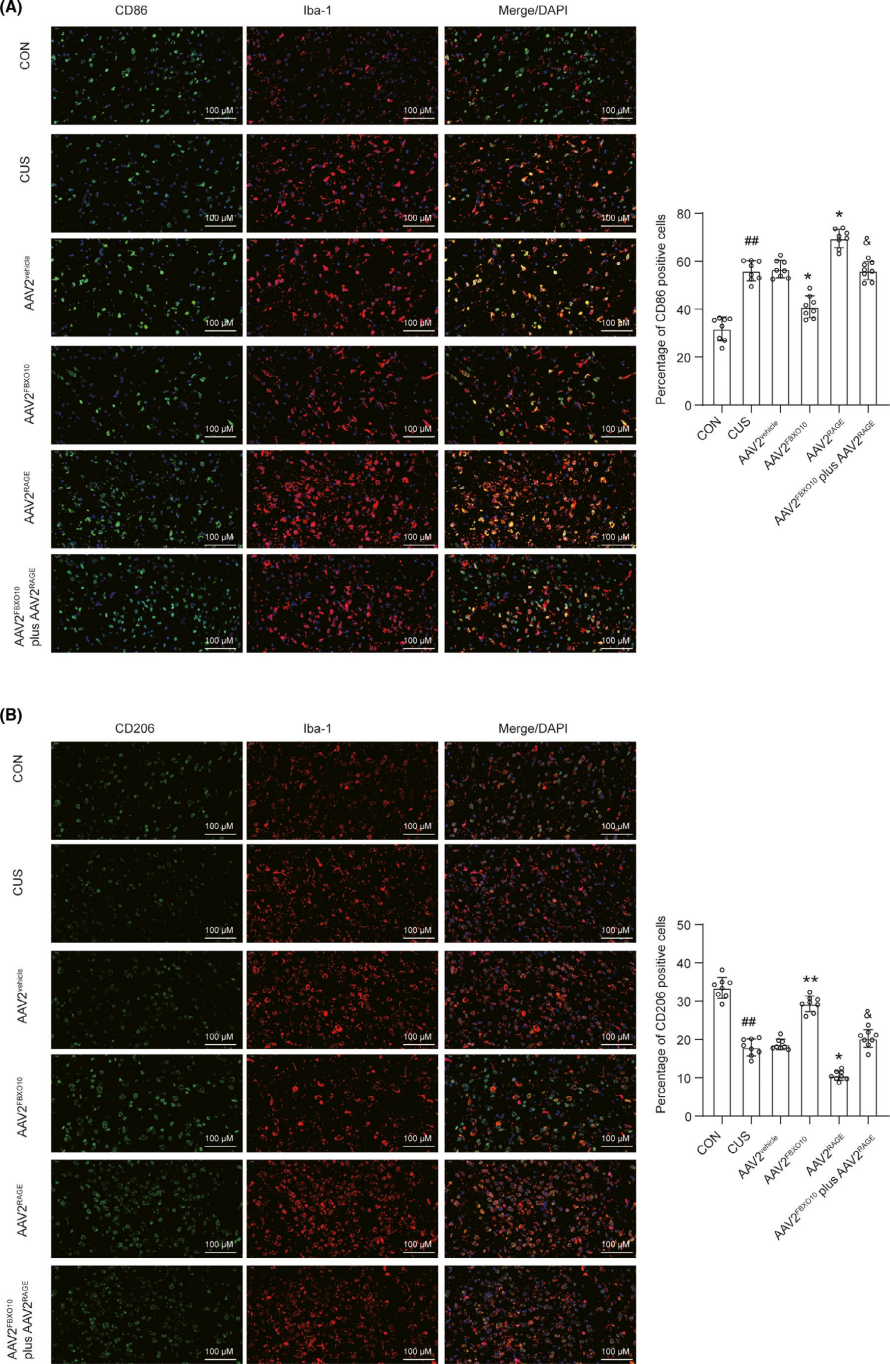
Co‐expression of Iba‐1 and M1/M2 phenotype markers. (A) Medial PFC co‐stained for Iba‐1 (microglia marker, red) and CD86 (M1 marker, green) and the quantification of CD86‐ and Iba‐1‐positive cells were shown in the right side (n = 8 per group). (B) Medial PFC co‐stained for Iba‐1 (red) and CD206 (M2 marker) (green) and the quantification of CD206‐ and Iba‐1‐positive cells were shown in the right side (n = 8 per group). Bars represent the mean ±SEM. ##*P *< 0.01 vs. control mice; *P < 0.05, **P < 0.01 vs. AAV2^vehicle^; &*P *< 0.05 vs. AAV2^FBXO10^

Based on the research above, FBXO10/RAGE axis contributed to preventing neuroinflammation induced by CUS. The proinflammatory (M1) and anti‐inflammatory (M2) activity of microglia play important role in the progression of neuroinflammation. We further analyzed the effects of FBXO10/RAGE axis on the expression of M1 markers (CD86) and M2 markers (CD206) by immunofluorescence. CUS exposure increased the immunofluorescence intensity of CD86 and decreased the immunofluorescence intensity of CD206 in the medial PFC, suggesting that CUS exposure promoted the accumulation of M1 phenotypic microglia (Figure 7A and B). RAGE overexpression further accelerated M1 phenotypic polarization of microglia induced by CUS exposure. FBXO10 overexpression obviously decreased CD86‐positive microglia and increased CD206‐positive microglia, indicating that FBXO10 promoted M1 phenotypic microglia toward M2 phenotype microglia in CUS mice. Furthermore, RAGE overexpression recovered the M2 phenotypic polarization of microglia induced by FBXO10 overexpression. Our results indicate that FBXO10/RAGE axis may improve CUS by regulating the polarization of microglia.

### 3.8 FBXO10/RAGE axis participates in LPS‐induced microglia polarization *in vitro*

**FIGURE 8:**
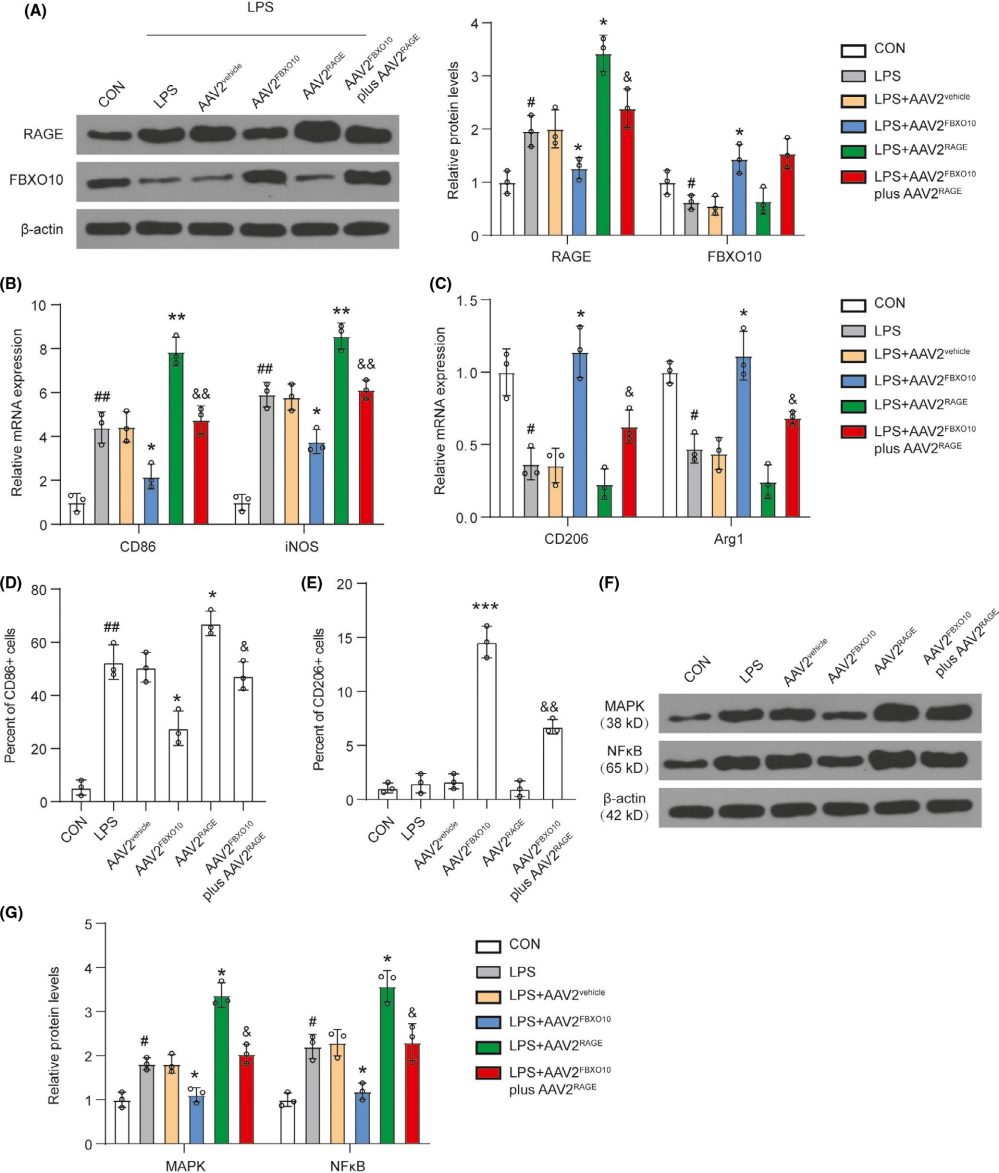
FBXO10/RAGE axis participates in LPS‐induced microglia polarization *in vitro*. BV2 cells with or without infection were treated with LPS for 16 h. (A) The protein levels of FBXO10 and RAGE in BV2 cells were detected by Western blotting. (B) The mRNA levels of M1 markers (CD86 and iNOS) were detected by qRT‐PCR. (C) The mRNA levels of M2 markers (CD206 and Arg1) were detected by qRT‐PCR. (D‐E) Surface expressions of CD86 and CD206 in BV2 cells after indicated treatment were detected by flow cytometry. The percentage of CD86+ and CD206+ cells in the microglia was determined. (F‐G) The protein levels of p38 MAPK and NF‐κΒ in BV2 cells were detected by Western blotting. Bars represent the mean ±SEM. #*P *< 0.05, ##*P *< 0.01 vs. control group; *P < 0.05, **P < 0.05, ***P < 0.01 vs. AAV2^vehicle^; &*P *< 0.05, &&*P *< 0.01 vs. AAV2^FBXO10^; n = 8 per group

To verify whether FBXO10/RAGE axis directly regulates M1/M2 polarization of microglia, BV2 cells were selected and activated by using 100 ng/ml LPS for 16 h stimulation. LPS stimulation upregulated RAGE protein levels and decreased FBXO10 protein levels (Figure 8A). LPS stimulation caused a robust increase in mRNA levels of M1 markers (CD86 and iNOS) in BV2 microglia cells (Figure 8B). FBXO10 overexpression significantly attenuated CD86 and iNOS mRNA expressions while RAGE overexpression significantly promoted mRNA levels of CD86 and iNOS after LPS treatment (Figure 8B). Under LPS treatment, RAGE overexpression recovered mRNA levels of CD86 and iNOS reduced by FBXO10 overexpression in BV2 microglia cells. Inversely, LPS treatment caused a decrease in mRNA levels of M2 markers (CD206 and Arg1) in BV2 microglia cells (Figure 8C). FBXO10 overexpression enhanced mRNA levels of CD206 and Arg1. RAGE overexpression recovered mRNA levels of CD206 and Arg1 enhanced by FBXO10 overexpression in BV2 microglia cells. In addition, the flow cytometry results showed an enhancement of the percentage of CD86+ cells and no significant change in the percentage of CD206+ cells after LPS treatment (Figure 8D and E). FBXO10 overexpression decreased the percentage of CD86+ cells and increased the percentage of CD206+ cells. RAGE overexpression recovered the effect on the percentage of CD86+ cells and CD206+ cells induced by FBXO10 overexpression. Moreover, p38 MAPK and NF‐κΒ were accumulated in BV2 microglia cells after LPS stimulation (Figure 8F and G). FBXO10 overexpression decreased p38 MAPK and NF‐κΒ protein expression whereas RAGE overexpression enhanced p38 MAPK and NF‐κΒ protein expression. RAGE overexpression reversed the accumulation of p38 MAPK and NF‐κΒ induced by FBXO10 overexpression, suggesting p38 MAPK and NF‐κΒ were the downstream effect factors for FBXO10/RAGE axis. All these results suggested that FBXO10/RAGE axis participated in microglia polarization.

## 4 DISCUSSION

The present study suggested that CUS exposure caused an increase of RAGE, a decrease of FBXO10, the abnormal expression of inflammatory cytokine and neurotrophic factor BDNF in PFC. Importantly, CUS exposure induced M1 phenotypic polarization of microglia. FBXO10 overexpression or RAGE knockdown inhibited proinflammatory cytokine release, promoted BDNF expression, mitigated the depressive‐like and cognitive impairment behaviors, and inhibited the polarization of M1 microglia. We also identified that p38 MAPK and NF‐κΒ were the downstream effect factors for FBXO10/RAGE axis. Our study may provide new targets for the treatment of depression.

A growing body of research shows that chronic stress exposure causes PFC dysfunction which is involved in the neurobiology of depression.^17^, ^18^ Stress exposure induces morphological and functional alterations in microglia which participates in homeostatic neuronal function and synaptic plasticity.^19^, ^20^, ^21^, ^22^ Stress‐induced neuronal remodeling is reported to be attributed to resident microglia.^2^, ^23^ In this study, we found that CUS exposure caused depressive‐like behaviors and cognitive impairment. These behavioral effects were related to the dysregulated microglia function, including altered expression of neuroinflammation and the polarization of microglia in the medial PFC.

Neuroinflammation plays a crucial role in affective disorders progression^24^ and could be a contributing factor in the development of depression.^25^, ^26^ The activation of microglia^27^, ^28^ and overexpression of proinflammatory cytokines in some brain areas are associated with the development of depression.^29^, ^30^, ^31^ Peripheric inflammation activation such as the increased levels of IL‐1β, IL‐6, and interferon‐γ are found in depressive patients or animal model of depression.^32^, ^33^ Activated microglia has been regarded as a key source of local proinflammatory cytokines in the brain, including IL‐1β, IL‐18, TNFα, and IL‐6, driving progressive neuron damage.^34^Treatment with IL‐1β receptor antagonist reverses CUS‐induced depressive‐like behavior.^16^, ^35^ Our results showed that after CUS exposure, TNFα and IL‐1β were increased whereas TGFβ were decreased. However, there was no significant change for IL‐6 and IL‐10 in CUS‐induced mice. In this process, Iba‐1, the activated marker of microglia was consistently increased, indicating that microglia were activated in PFC following chronic stress.

The polarization balance of microglia is a hot topic in recent years. Microglia actively maintain central nervous system integrity and regulate brain functions.^36^ The polarization of microglia was closely related to brain disorders and cognitive impairment,^37^ brain recovery from neuronal injuries,^38^ as well as depression.^39^, ^40^, ^41^ As the polarization balance of M1/M2 can be reversed,^42^ treatment strategies based on regulating M1‐to‐M2 phenotypic conversion of microglial may be utilized in the treatment of depression. In the present study, we found that FBXO10/RAGE axis was involved in the polarization of microglia. FBXO10 overexpression decreased M1 phenotypic microglial and increased M2 phenotypic microglial. RAGE overexpression increased M1 phenotypic microglial and decreased M2 phenotypic microglial. These results suggested that FBXO10/RAGE axis promoted M1 to M2 phenotypic conversion.

In summary, we confirmed that FBXO10 triggered degradation of RAGE protein by ubiquitination also was involved in depression. We firstly explored the role of FBXO10 in CUS‐induced mice. FBXO10 administration prevented CUS‐induced behavioral despair, cognitive impairment, neuroinflammation, and the polarization microglia through decreasing the accumulation of RAGE, p38 MAPK, and NF‐κΒ. This work provides insight into mechanisms that link PFC dysfunction and associated behavioral consequences. Moreover, our data support the development of therapeutic strategies for treatment of psychiatric or neurological disease.

## CONFLICT OF INTEREST

JL, QZ, WS, MS, MX, and LM report no conflicts of interest.

## Supporting information

Fig S1‐S8

Supplementary Material

## Data Availability

The data that support the findings of this study are available from the corresponding author upon reasonable request.

## References

[1] Malhi GS , Mann JJ . Depression. Lancet. 2018;392(10161):2299‐2312. 30396512 10.1016/S0140-6736(18)31948-2

[2] Wohleb ES , Terwilliger R , Duman CH , Duman RS . Stress‐induced neuronal colony stimulating factor 1 provokes microglia‐mediated neuronal remodeling and depressive‐like behavior. Biol Psychiatry. 2018;83(1):38‐49. 28697890 10.1016/j.biopsych.2017.05.026 PMC6506225

[3] Kang HJ , Voleti B , Hajszan T , et al. Decreased expression of synapse‐related genes and loss of synapses in major depressive disorder. Nat Med. 2012;18(9):1413‐1417. 22885997 10.1038/nm.2886 PMC3491115

[4] Shansky RM , Hamo C , Hof PR , McEwen BS , Morrison JH . Stress‐induced dendritic remodeling in the prefrontal cortex is circuit specific. Cereb Cortex. 2009;19(10):2479‐2484. 19193712 10.1093/cercor/bhp003 PMC2742599

[5] Horchar MJ , Wohleb ES . Glucocorticoid receptor antagonism prevents microglia‐mediated neuronal remodeling and behavioral despair following chronic unpredictable stress. Brain Behav Immun. 2019;81:329‐340. 31255679 10.1016/j.bbi.2019.06.030

[6] Yang F , Wang H , Chen H , et al. RAGE Signaling pathway in hippocampus dentate gyrus involved in GLT‐1 decrease induced by chronic unpredictable stress in rats. Brain Res Bull. 2020;163:49‐56. 32621862 10.1016/j.brainresbull.2020.06.020

[7] Han SH , Kim YH , Mook‐Jung I . RAGE: the beneficial and deleterious effects by diverse mechanisms of actions. Mol Cells. 2011;31(2):91‐97. 21347704 10.1007/s10059-011-0030-x PMC3932687

[8] Origlia N , Criscuolo C , Arancio O , Yan SS , Domenici L . RAGE inhibition in microglia prevents ischemia‐dependent synaptic dysfunction in an amyloid‐enriched environment. J Neurosci. 2014;34(26):8749‐8760. 24966375 10.1523/JNEUROSCI.0141-14.2014 PMC4069353

[9] Franklin TC , Wohleb ES , Zhang Y , Fogaca M , Hare B , Duman RS . Persistent increase in microglial RAGE contributes to chronic stress‐induced priming of depressive‐like behavior. Biol Psychiatry. 2018;83(1):50‐60. 28882317 10.1016/j.biopsych.2017.06.034 PMC6369917

[10] Adamopoulos C , Piperi C , Gargalionis AN , et al. Advanced glycation end products upregulate lysyl oxidase and endothelin‐1 in human aortic endothelial cells via parallel activation of ERK1/2‐NF‐kappaB and JNK‐AP‐1 signaling pathways. Cell Mol Life Sci. 2016;73(8):1685‐1698. 26646068 10.1007/s00018-015-2091-z PMC11108501

[11] Chiorazzi M , Rui L , Yang Y , et al. Related F‐box proteins control cell death in Caenorhabditis elegans and human lymphoma. Proc Natl Acad Sci U S A. 2013;110(10):3943‐3948. 23431138 10.1073/pnas.1217271110 PMC3593917

[12] Evankovich J , Lear T , McKelvey A , et al. Receptor for advanced glycation end products is targeted by FBXO10 for ubiquitination and degradation. FASEB J. 2017;31(9):3894‐3903. 28515150 10.1096/fj.201700031R PMC5572686

[13] Li Y , Bouchlaka MN , Wolff J , et al. FBXO10 deficiency and BTK activation upregulate BCL2 expression in mantle cell lymphoma. Oncogene. 2016;35(48):6223‐6234. 27157620 10.1038/onc.2016.155 PMC5102814

[14] Guo F , Luo Y , Jiang X , et al. Recent BCR stimulation induces a negative autoregulatory loop via FBXO10 mediated degradation of HGAL. Leukemia. 2020;34(2):553‐566. 31570756 10.1038/s41375-019-0579-5

[15] Wang D , Liu F , Zhu L , et al. FGF21 alleviates neuroinflammation following ischemic stroke by modulating the temporal and spatial dynamics of microglia/macrophages. J Neuroinflammation. 2020;17(1):257. 32867781 10.1186/s12974-020-01921-2 PMC7457364

[16] Yue N , Li B , Yang L , et al. Electro‐acupuncture alleviates chronic unpredictable stress‐induced depressive‐ and anxiety‐like behavior and hippocampal neuroinflammation in rat model of depression. Front Mol Neurosci. 2018;11:149. 29946236 10.3389/fnmol.2018.00149 PMC6007169

[17] Holmes SE , Scheinost D , Finnema SJ , et al. Lower synaptic density is associated with depression severity and network alterations. Nat Commun. 2019;10(1):1529. 30948709 10.1038/s41467-019-09562-7 PMC6449365

[18] Wohleb ES , Franklin T , Iwata M , Duman RS . Integrating neuroimmune systems in the neurobiology of depression. Nat Rev Neurosci. 2016;17(8):497‐511. 27277867 10.1038/nrn.2016.69

[19] Wu Y , Dissing‐Olesen L , MacVicar BA , Stevens B . Microglia: dynamic mediators of synapse development and plasticity. Trends Immunol. 2015;36(10):605‐613. 26431938 10.1016/j.it.2015.08.008 PMC4841266

[20] Yirmiya R , Rimmerman N , Reshef R . Depression as a microglial disease. Trends Neurosci. 2015;38(10):637‐658. 26442697 10.1016/j.tins.2015.08.001

[21] Schafer DP , Lehrman EK , Kautzman AG , et al. Microglia sculpt postnatal neural circuits in an activity and complement‐dependent manner. Neuron. 2012;74(4):691‐705. 22632727 10.1016/j.neuron.2012.03.026 PMC3528177

[22] Hong S , Beja‐Glasser VF , Nfonoyim BM , et al. Complement and microglia mediate early synapse loss in Alzheimer mouse models. Science. 2016;352(6286):712‐716. 27033548 10.1126/science.aad8373 PMC5094372

[23] Frost JL , Schafer DP . Microglia: architects of the developing nervous system. Trends Cell Biol. 2016;26(8):587‐597. 27004698 10.1016/j.tcb.2016.02.006 PMC4961529

[24] Eyre H , Baune BT . Neuroimmunological effects of physical exercise in depression. Brain Behav Immun. 2012;26(2):251‐266. 21986304 10.1016/j.bbi.2011.09.015

[25] Kopschina Feltes P , de Vries EF , Juarez‐Orozco LE , et al. Repeated social defeat induces transient glial activation and brain hypometabolism: A positron emission tomography imaging study. J Cereb Blood Flow Metab. 2019;39(3):439‐453. 29271288 10.1177/0271678X17747189 PMC6399731

[26] Wang H , Zhao Y , Wang YJ , et al. Antidepressant‐like effects of tetrahydroxystilbene glucoside in mice: Involvement of BDNF signaling cascade in the hippocampus. CNS Neurosci Ther. 2017;23(7):627‐636. 28547794 10.1111/cns.12708 PMC6492667

[27] Takizawa T , Shibata M , Kayama Y , et al. High‐mobility group box 1 is an important mediator of microglial activation induced by cortical spreading depression. J Cereb Blood Flow Metab. 2017;37(3):890‐901. 27142867 10.1177/0271678X16647398 PMC5363469

[28] Shibata M , Suzuki N . Exploring the role of microglia in cortical spreading depression in neurological disease. J Cereb Blood Flow Metab. 2017;37(4):1182‐1191. 28155572 10.1177/0271678X17690537 PMC5414895

[29] Yue N , Huang H , Zhu X , et al. Activation of P2X7 receptor and NLRP3 inflammasome assembly in hippocampal glial cells mediates chronic stress‐induced depressive‐like behaviors. J Neuroinflammation. 2017;14(1):102. 28486969 10.1186/s12974-017-0865-y PMC5424302

[30] Rinwa P , Kumar A . Quercetin suppress microglial neuroinflammatory response and induce antidepressent‐like effect in olfactory bulbectomized rats. Neuroscience. 2013;255:86‐98. 24095694 10.1016/j.neuroscience.2013.09.044

[31] Hu W , Zhang Y , Wu W , et al. Chronic glucocorticoids exposure enhances neurodegeneration in the frontal cortex and hippocampus via NLRP‐1 inflammasome activation in male mice. Brain Behav Immun. 2016;52:58‐70. 26434621 10.1016/j.bbi.2015.09.019

[32] Miller AH , Maletic V , Raison CL . Inflammation and its discontents: the role of cytokines in the pathophysiology of major depression. Biol Psychiatry. 2009;65(9):732‐741. 19150053 10.1016/j.biopsych.2008.11.029 PMC2680424

[33] Zhang Y , Liu L , Liu YZ , et al. NLRP3 inflammasome mediates chronic mild stress‐induced depression in mice via neuroinflammation. Int J Neuropsychopharmacol. 2015;18(8). 10.1093/ijnp/pyv006 PMC4571628 25603858

[34] Song C , Wang H . Cytokines mediated inflammation and decreased neurogenesis in animal models of depression. Prog Neuropsychopharmacol Biol Psychiatry. 2011;35(3):760‐768. 20600462 10.1016/j.pnpbp.2010.06.020

[35] Koo JW , Duman RS . IL‐1beta is an essential mediator of the antineurogenic and anhedonic effects of stress. Proc Natl Acad Sci U S A. 2008;105(2):751‐756. 18178625 10.1073/pnas.0708092105 PMC2206608

[36] Xie D , He M , Hu X . Microglia/macrophage diversities in central nervous system physiology and pathology. CNS Neurosci Ther. 2019;25(12):1287‐1289. 31793210 10.1111/cns.13257 PMC7154592

[37] Shi L , Rocha M , Zhang W , et al. Genome‐wide transcriptomic analysis of microglia reveals impaired responses in aged mice after cerebral ischemia. J Cereb Blood Flow Metab. 2020;40(1_suppl):S49‐S66. 32438860 10.1177/0271678X20925655 PMC7687039

[38] Hu X . Microglia/macrophage polarization: Fantasy or evidence of functional diversity? J Cereb Blood Flow Metab. 2020;40(1_suppl):S134‐S136. 33023387 10.1177/0271678X20963405 PMC7687031

[39] Tang J , Yu W , Chen S , Gao Z , Xiao B . Microglia polarization and endoplasmic reticulum stress in chronic social defeat stress induced depression mouse. Neurochem Res. 2018;43(5):985‐994. 29574669 10.1007/s11064-018-2504-0

[40] Kalkman HO , Feuerbach D . Antidepressant therapies inhibit inflammation and microglial M1‐polarization. Pharmacol Ther. 2016;163:82‐93. 27101921 10.1016/j.pharmthera.2016.04.001

[41] Gu XH , Xu LJ , Zheng LL , et al. Long non‐coding RNA uc.80‐ overexpression promotes M2 polarization of microglias to ameliorate depression in rats. IUBMB Life. 2020;72(10):2194‐2203. 32780551 10.1002/iub.2353

[42] Ji J , Xue TF , Guo XD , et al. Antagonizing peroxisome proliferator‐activated receptor gamma facilitates M1‐to‐M2 shift of microglia by enhancing autophagy via the LKB1‐AMPK signaling pathway. Aging Cell. 2018;17(4):e12774. 29740932 10.1111/acel.12774 PMC6052482

