# Supplementary material for: FBXO10 prevents chronic unpredictable stress‐induced behavioral despair and cognitive impairment through promoting RAGE degradation
Source: CNS Neurosci Ther. 2021 Sep 7;27(12):1504–17. doi: 10.1111/cns.13727 (PMC8611766; doi:10.1111/cns.13727)
Supplement: Supplementary file 1 — Fig S1‐S8 [file CNS-27-1504-s001.pdf]

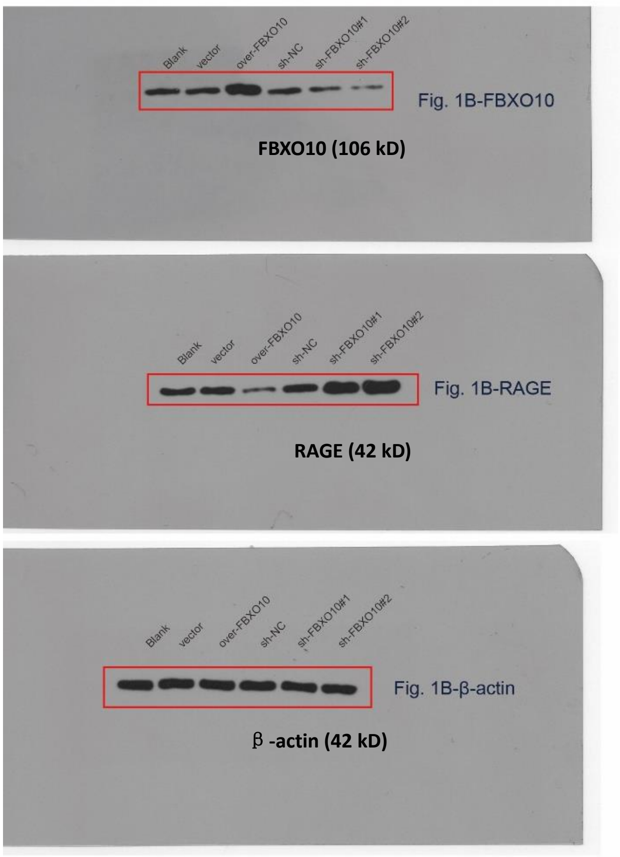

Full unedited blot for Figure 1B

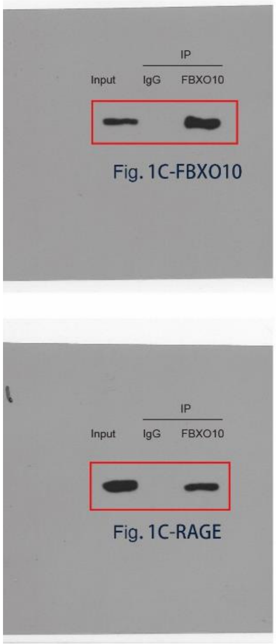

FBXO10 (106 kD)

RAGE (42 kD)

Full unedited blot for Figure 1C

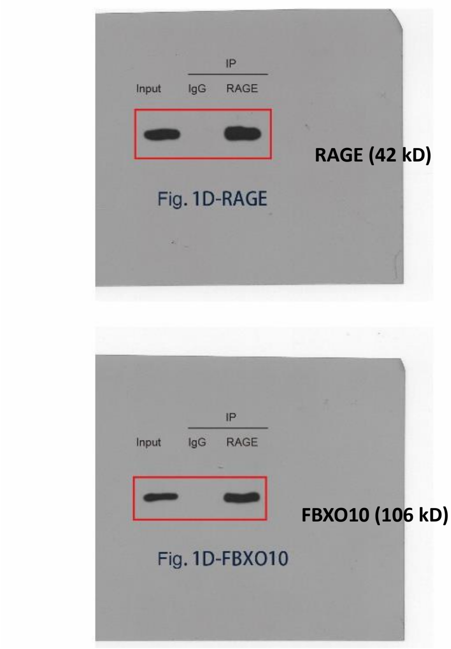

Full unedited blot for Figure 1D

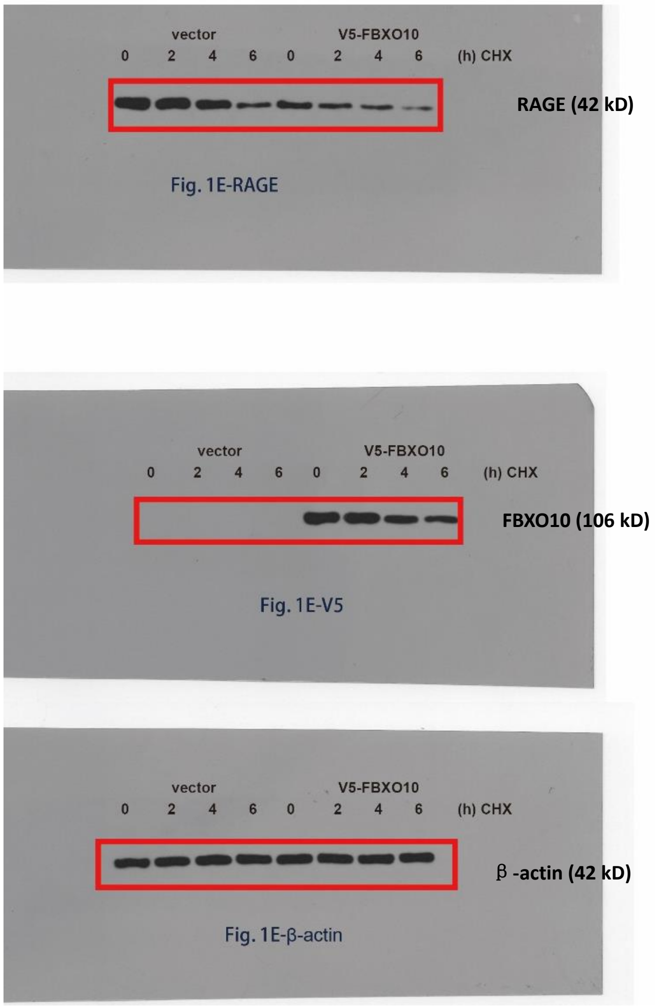

Full unedited blot for Figure 1E

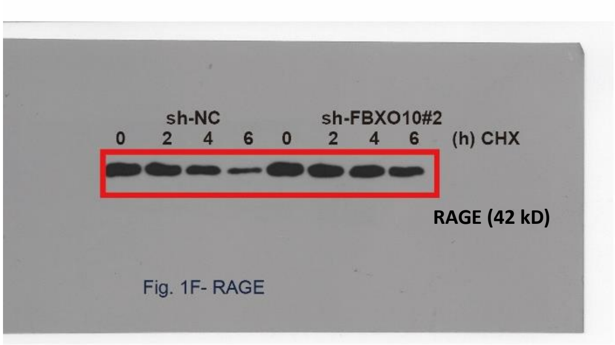

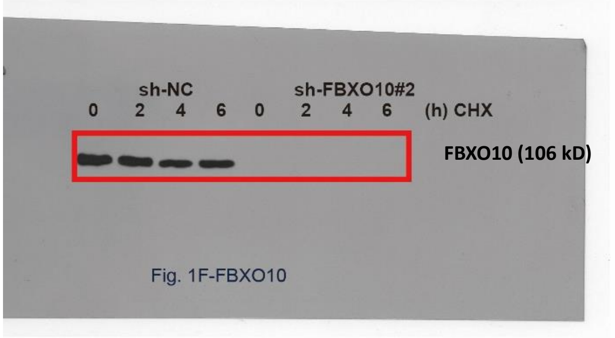

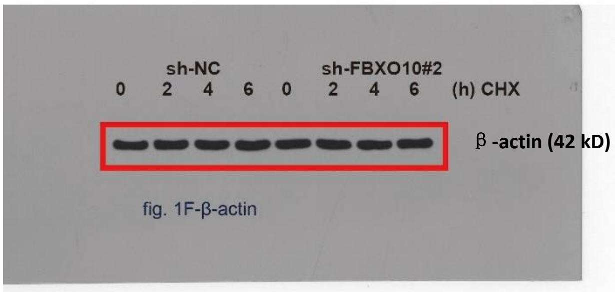

Full unedited blot for Figure 1F

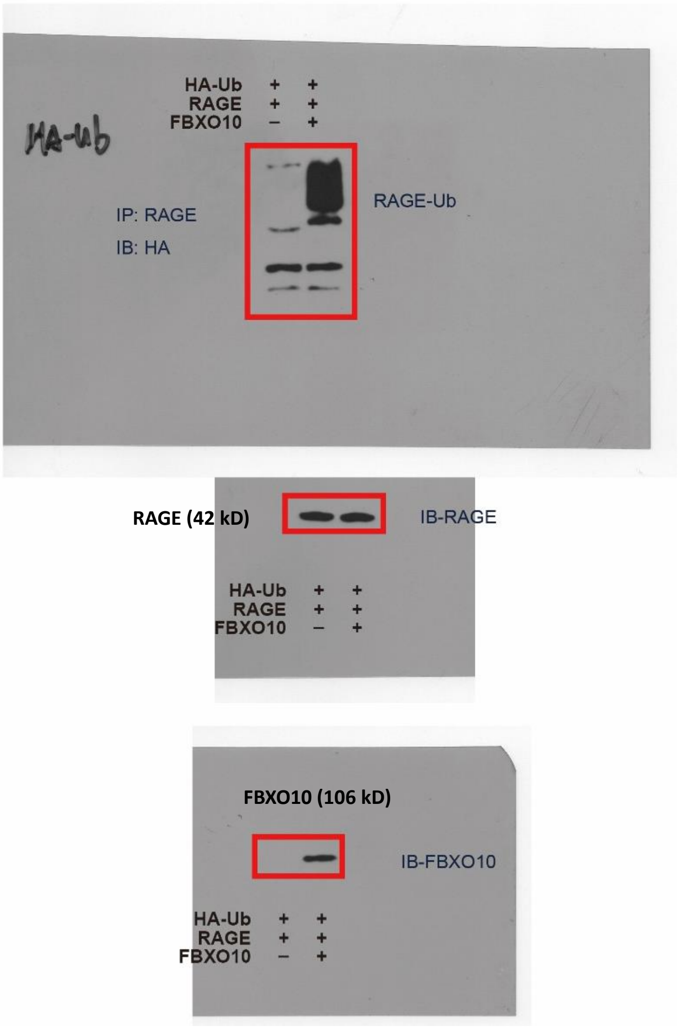

Full unedited blot for Figure 1G

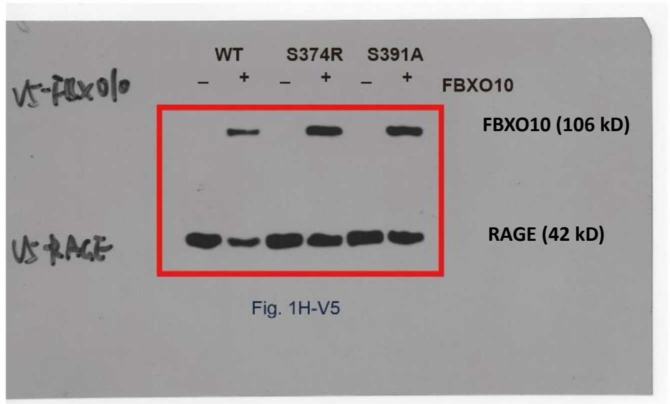

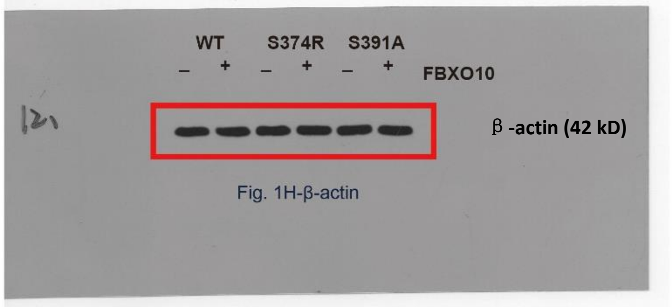

Full unedited blot for Figure 1H

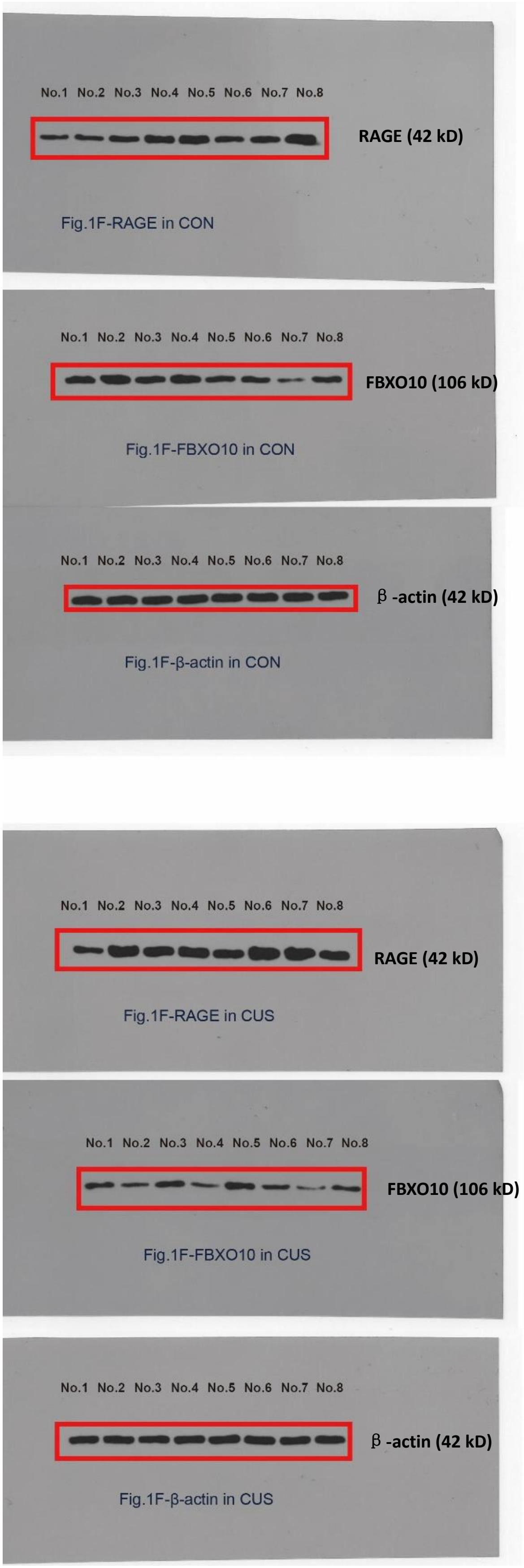

Full unedited blot for Figure 2F

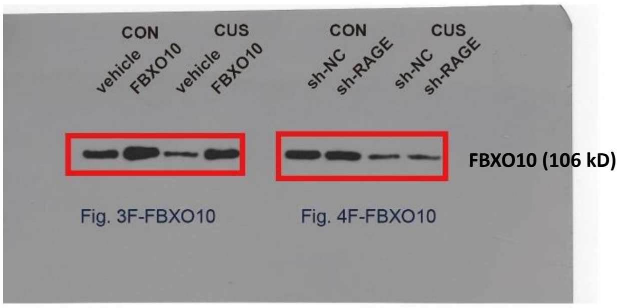

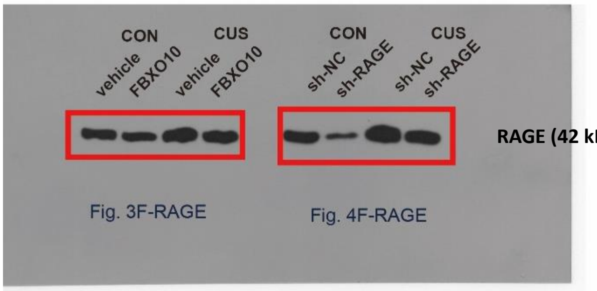

RAGE (42 kD)

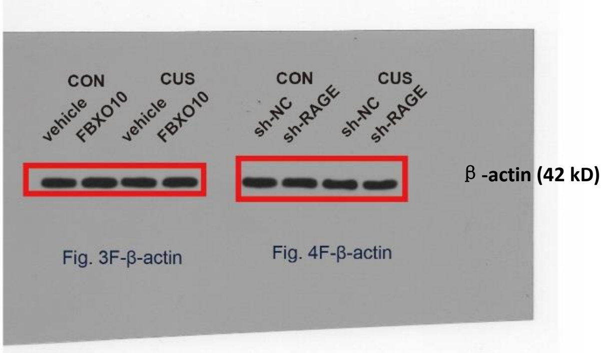

Full unedited blot for Figure 3F and 4F

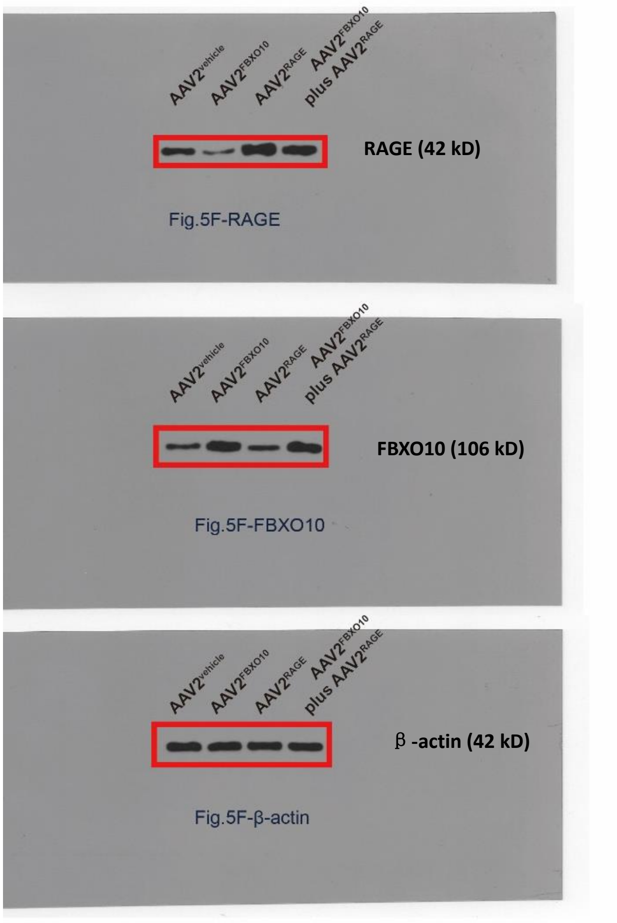

Full unedited blot for Figure 5F

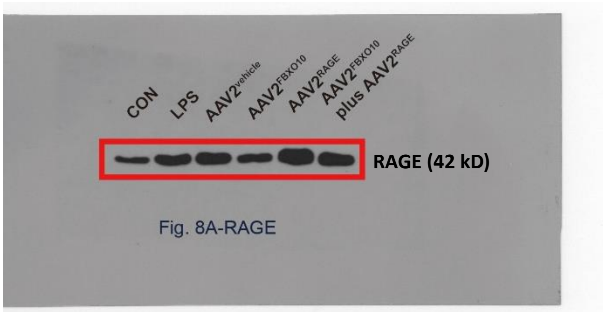

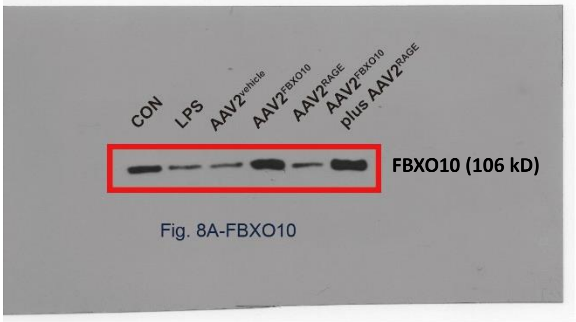

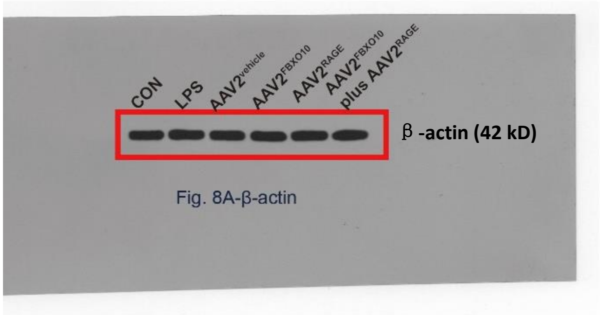

Full unedited blot for Figure 8A

RAGE (42 kD)

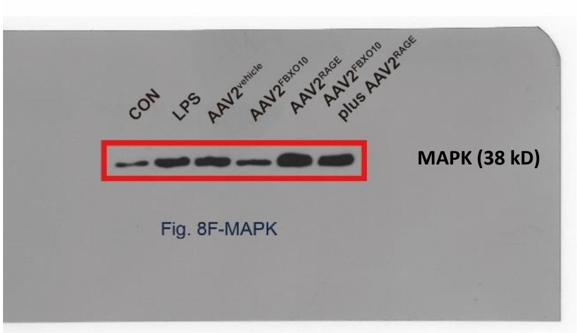

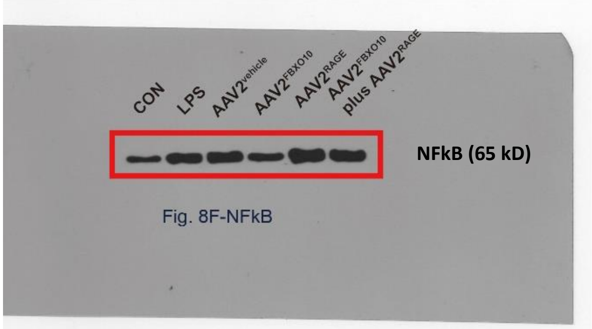

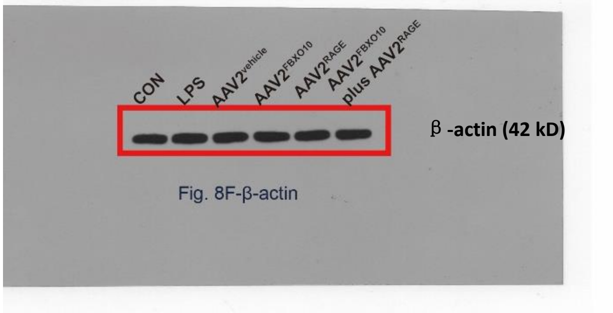

Full unedited blot for Figure 8F
