# Supplementary material for: FBXO10 prevents chronic unpredictable stress‐induced behavioral despair and cognitive impairment through promoting RAGE degradation
Source: CNS Neurosci Ther. 2021 Sep 7;27(12):1504–17. doi: 10.1111/cns.13727 (PMC8611766; doi:10.1111/cns.13727)
Supplement: Supplementary file 2 — Supplementary Material [file CNS-27-1504-s002.docx]

**Supplemental Methods**

**Viral preparation**

To enhance FBXO10 or RAGE exogenous expressions, total full-length sequences of FBXO10 or RAGE was inserted into an AAV2 plasmid under transcriptional regulation of CMV promoter. The [specific](javascript:;) short-hairpin RNA (shRNA) of FBXO10 or RAGE were synthesized by GenePharma. The shRNA for RAGE was ligated into the AAV2 plasmid. Construct was packaged in adeno-associated virus-2 (AAV2) as previously described [1,2].

**Behavioral Testing**

For the FST, mice were placed in a 2-liter beaker of water (24°+/- 1 °C) for ten minutes and time spent immobile was determined.

For the SCT, the night before testing bottles containing water were placed in cage overnight to measure baseline water consumption. In the morning water bottles were removed and mice were water-deprived for 12 h. Bottles containing water with 1% sucrose were placed in the cage for 1 h and then weighed for sucrose consumption.

For the NSF, mice were placed in a white plastic arena (22 × 30 × 14 cm) with fresh cage bedding covering the bottom. Three small pellets of mouse chow were placed in the center of the arena and a blinded observer recorded their latency to feed.

For the TOR, mice were placed in a plastic arena with two plastic Lego™ trees secured to the bottom of the arena for five minutes. Thirty minutes after the initial stage, mice were placed in the same arena with Lego™ blocks for five minutes. An hour after the second stage, the mice were placed in the arena with one block and one tree, counterbalanced to prevent bias. The time spent exploring each of these objects was measured. The discrimination index was calculated: difference between time spent exploring the tree or the block divided by the total time spent exploring the tree and the block.

**Western blot**

The total protein in medial PFC of mice and BV2 cells were obtained by using RIPA buffer (Thermo Scientific). Protein samples were dispersed on 12% tris-glycine SDS-PAGE gels and transferred to PVDF membrane. After incubated with primary antibody against FBXO10 (Thermo Scientific, PA5-113506), RAGE (Abcam, ab172473), NF-κΒ (Abcam, ab32536), p38 MAPK (Abcam, ab182453) and β-actin (Abcam, ab8227) for overnight at 4 °C, secondary antibodies (1:20000, Abcam) were incubated for 1 h at room temperature. The relative proteins levels were detected by enhanced chemiluminescence reagent and ImageJ software.

**RNA isolation and real-time PCR**

RNA was extracted from medial PFC of mice using TRIzol Reagent. Samples were reverse transcribed, and real-time PCR conducted as previously described [3]. Oligonucleotide primers specific for CD86 iNOS CD206 and Arg1 were listed as followed. CD86 forward 5’-TAGGGATAACCAGGCTCTAC-3’, reverse 5’-CGTGGGTGTCTTTTGCTGTA-3’; iNOS forward 5’-CAAGCACCTTGGAAGA GGAG-3’, reverse 5’-AAGGCCAAACACAGCATACC-3’; CD206 forward 5’-AGTTGGGTTCTCCTGTAGCCCAA-3’, reverse 5’-ACTACTACCTGAGCCC ACACCTGCT-3’; Arg1 forward 5’-TCACCTGAGCTTTGATGTCG-3’, reverse 5’-CTGAAAGGAGCCCTGTCTTG-3’; FBXO10 forward 5’-ATGTGGCGTGTGG ATGATGT-3’, reverse 5’-CCAGGGAACTCGCCATCTTT-3’; RAGE forward 5’-ACATGTGTGTCTGAGGGAAGC-3’, reverse 5’-AGCTCTGACCGCAGTGT AAAG-3’; β-actin forward 5’-CACTGCAAACGGGGAAATGG-3’, reverse 5’-TGAGATGGACTGTCGGATGG-3’.

**ELISA of brain homogenate**

The inflammatory cytokines TNF-α, IL-1β, IL-6, IL-10 and TGF-β and the neurotrophic factor BDNF in tissue homogenate of medial PFC were measured using commercially available ELISA kits (FineTest, China).

**Protein Half-Life Detection**

BV2 cells were separately transfected with sh-NC, sh- sh-FBXO10#2, V5-FBXO10 or vector plasmids and treated with 100 μg/mL cycloheximide (CHX). after culturing for 0, 2, 4 and 6 hours, the treated cells were collected for protein extraction and Western blotting. A total of 20 μg protein was loaded and anti-RAGE, anti-FBXO10 or anti-V5 was used as the primary antibody. Relative expression of RAGE (normalized toβ-actin protein expression) was evaluated using ImageJ software.

***In vitro* ubiquitination assays**

*In vitro* ubiquitination assays were also performed to further prove that FBXO10 regulate RAGE ubiquitination directly. FBXO10 was overexpressed in BV2 cells and the FBXO10 complex was affinity-purified using anti-FBXO10 antibody and then incubated with purified RAGE protein in the in vitro ubiquitination reaction (R&D Systems). Then, Western blot assays were used to detect the ubiquitination of the RAGE protein subsequently.

**References**

1. **Ota KT, Liu RJ, Voleti B, Maldonado-Aviles JG, Duric V, Iwata M, Dutheil S, Duman C, Boikess S, Lewis DA, Stockmeier CA, DiLeone RJ, Rex C, Aghajanian GK, Duman RS.** REDD1 is essential for stress-induced synaptic loss and depressive behavior. *Nat Med*. 2014; 20: 531-5.

2. **Wohleb ES, Terwilliger R, Duman CH, Duman RS.** Stress-Induced Neuronal Colony Stimulating Factor 1 Provokes Microglia-Mediated Neuronal Remodeling and Depressive-like Behavior. *Biol Psychiatry*. 2018; 83: 38-49.

3. **Yue N, Huang H, Zhu X, Han Q, Wang Y, Li B, Liu Q, Wu G, Zhang Y, Yu J.** Activation of P2X7 receptor and NLRP3 inflammasome assembly in hippocampal glial cells mediates chronic stress-induced depressive-like behaviors. *J Neuroinflammation*. 2017; 14: 102.

**Supplementary Table S1 Daily sequence of the individual stressors for CUS**

| **Day** | **Daytime** | **Nighttime** |
| --- | --- | --- |
| **1** | Cage rotation | isolation |
| **2** | food deprivation | light on |
| **3** | crowding | no bedding |
| **4** | physical restraint | water deprivation |
| **5** | tilted cage | food deprivation |
| **6** | light off | water deprivation |
| **7** | physical restraint | light on |
| **8** | wet bedding | no bedding |
| **9** | crowding | stroboscope overnight |
| **10** | food deprivation | physical restraint |
| **11** | tilted cage | water deprivation |
| **12** | Forced swim test | Cage rotation |
| **13** | Sucrose consumption test | no bedding |
| **14** | Novelty-suppressed feeding test | physical restraint |
| **15** | Temporal object recognition task |  |
